# Simultaneous aortic and tricuspid valve rupture after fall injury

**DOI:** 10.5249/jivr.v5i2.455

**Published:** 2013-06

**Authors:** Feridoun Sabzi, Mojtaba Niazi, Alireza Ahmadi

**Affiliations:** ^*a*^ Imam Ali Heart Center, Kermanshah University of Medical Sciences, Kermanshah, Iran.; ^*b*^Department of Anesthesiology, Imam Reza Hospital, Kermanshah University of Medical Sciences, Kermanshah, Iran.

**Keywords:** Cardiac injury, Blunt trauma, Fall injury

## Abstract

This case study concerns a patient with disruption of both tricuspid and aortic valves: a previously healthy, adult man, who sustained a 5-meter fall from a building under construction. The mechanism of the injury was acceleration and deceleration, acting in two different phases of the cardiac cycle, i.e. systole and diastole. Simultaneous occurrence of these injuries is exceedingly rare and in a careful literature review, we did not find any such combination of injury. The possible mechanisms of this injury, as well as surgical techniques are discussed.

## Introduction

One of the most common injuries after blunt chest trauma is myocardial contusion,^1^ which itself is probably under-diagnosed. Septal and free-wall ruptures of all four chambers have been well described in postmortem series.^2^ Right ventricle is the most commonly ruptured chamber in closed-chest trauma and coronary artery disruption is rare.^2^ Further, postmortem series and the clinical literature suggest that acute valvular dysfunction after chest trauma is very rare. Blunt Trauma, especially involving fall from a multi floor building, can result in serious multi organ injuries. It is proposed that the underlying mechanism cause to these injuries requires a high amount of inertia released by the above mechanism in a fall from a high building.^3^ We report a case of rupture of both tricuspid and aortic valves in a previously healthy 34-year-old man who fell from a height of 5 meters and sustained blunt chest trauma.

## Case Study

**Figure 1:**
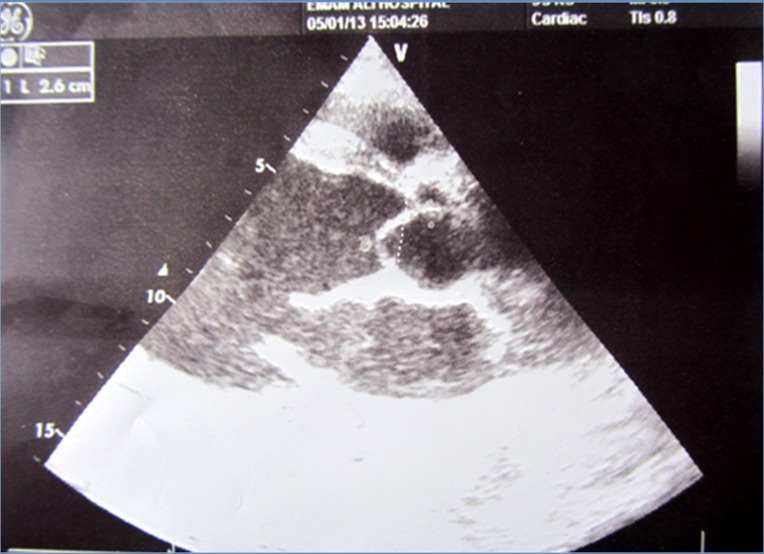
Transesophageal echocardiography revealing a flail aortic valve.

**Figure 2:**
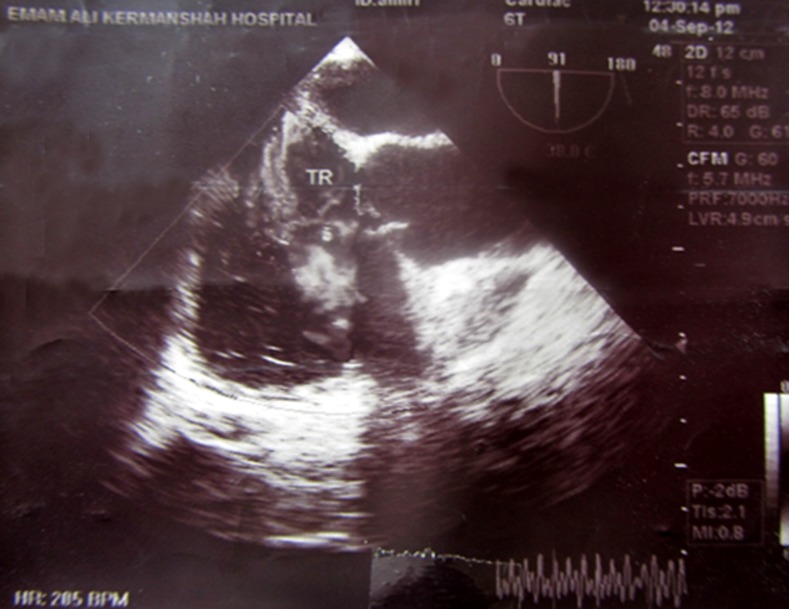
Transesophageal Doppler study revealing severe tricuspid regurgitation

A previously healthy 34-year-old construction laborer was admitted to our hospital emergency room from a local hospital complaining of multiple trauma and dyspnea and chest pain. He had fallen approximately 5 meters from the top floor of a new building under construction. He first hit a first floor balcony where he struck his sternum on a balcony ridge transversely. From there he fell to the mosaic bricks at the bottom of the building. He did not have skull fracture and did not lose consciousness. He was transported at once to the hospital where he developed acute dyspnea of breath and pain shortly after his fall. Upon arrival, he was awake and oriented with a heart rate of 120 beats /minute, weak peripheral pulses, reduced left hemithorax breath sounds, and an obvious contusion across his mid sternum and left rib cage. Other injuries including severe contusion and laceration of face and skull skin and left tibia and left radius were found. Shortly after his arrival at the emergency room, the patient became anxious with increasing dyspnea, and his condition rapidly deteriorated. Systolic blood pressure decreased from 90 mm Hg to 60 mm Hg, heart rate increased to 150 beats /minute, and peripheral pulse oximetry indicated significant oxyhemoglobin desaturation. Chest radiography revealed complete opacity of left hemithorax, large heart shadow and 3-6 fractured left ribs. The patient was intubated and copious, frothy, edema fluid was suctioned from the treacheal tube. Chest roentgenogram also revealed florid, right lung, pulmonary edema with progressive deterioration of patient’s hemodynamic, a presumptive diagnosis of the left hemithorax was made. Because of the immediately life-threatening nature of the patient’s problem, a left hemithorax chest tube was inserted and approximately 1000 milliliters of blood was immediately evacuated. However, the patient’s vital signs did not improve and a left anterior thoracotomy was performed in the operating room and a large amount of clotted blood, which was causing tension to the hemithorax, was evacuated. Exploration revealed transaction of the left internal mammary artery (LIMA) and intercostal arteries in 4th intercostals space. After the thoracotomy, both ends of LIMA were ligated and a chest tube was fixed. After initial resuscitation, his femoral and radius fractures were internally fixed and the patient transferred to ICU. Systolic and diastolic murmurs were subsequently heard when the patient was back at the ICU. Shortly before an episode of pulmonary edema, transthoracic echocardiography revealed a marked regurgitation of the aortic valve (due to a flail noncoronary cusp) and tricuspid regurgitation. (Figure 1, 2) The decision was made to proceed to surgical repair due to circulatory instability. On opening the pericardium, there was bruising of the aortic root and right atrial wall, but the heart was noted to be quite full and loaded and hyperdynamic. After establishing cardiopulmonary bypass (CPB) and cardioplegic arrest, the aorta was opened. The aortic valve had three leaflets with detachments of commeasure of the noncoronary and right coronary cusps that caused these two cusps to be flail. It was freely prolapsing into the left ventricular outflow tract. The commissural attachment was sutured back to the aortic wall with two pledgets 4-0 ethibond sutures. The aorta was closed with two running 4-0 prolene sutures. After aortic valve repair, the right atrium was opened and inspection of the tricuspid valve revealed that the anterior and posterior leaflets of the tricuspid valve were completely prolapsed into the right atrium since their papillary muscle support had severely ruptured and were edematous and did not accept any sutures. We decided to replace it with a 29 Carpentier-Edwards valve. The atrium was closed with two running 4-0 Viline sutures and the patient weaned from cardiopulmonary bypass using inotropic drugs. Transoesophageal echocardiography showed that the aortic valve was competent with trivial regurgitation.

The patient was extubated on the second postoperative day. Perioperative cardiac troponin level determination did not suggest the patient had a myocardial infarction. Neurologic examination was normal. The patient was discharged from the intensive care unit on the third postoperative day and home from the hospital on the eighth postoperative day in satisfactory condition. 

## Discussion

After a careful literature review, we discovered that this is probably the first single case report of simultaneous injury of both aortic and tricuspid valves that was associated with falling from height. Although cardiac valvular injuries are relatively uncommon after trauma, valvular damage has been repeatedly reported after severe trauma.^3^ The tricuspid valve is perhaps particularly vulnerable because the right ventricle is immediately behind the sternum.^4^ However, it is unclear as to whether any particular cardiac valve is more prone to injury from trauma.^5^ As opposed to tricuspid injury, aortic valve tear following blunt chest trauma is very rare.^6^ About only 100 cases have been reported all over the world up to 2002.^7^ Our case report has two unique features: firstly the simultaneous combination of double valve injury; and secondly, each valve injury has different induced mechanism. The mechanism causing aortic valve rupture is considered to be a sudden increase in intra-aortic pressure during a vulnerable phase of the cardiac cycle, especially during diastole, when the aortic valve is closed and the trans-aortic gradient is maximal.^6^ This high pressure caused detachment of the commissur or a tear or rupture of the aortic cusp. Non-coronary cusp is most commonly involved.^8^ In Asbach’s study the detachment of the commissur and twenty-eight aortic cusp lesions were described. Fourteen were noncoronary, 10 right coronary and only 4 left coronary.^9^ In contrast to the aortic valve injury that occurs during diastole, Gayet and colleagues reported that the purported mechanism of tricuspid injury is compression of the heart during isovolemic systole.^10^ Liedtke revealed that at this time the cardiac chambers are fully loaded and the valves are closing or closed.^11^ Acute, significant thoracic compression transmits pressure to intra-ventricular space and probably leads to severe tricuspid valve prolapse and rupture by disruption at the chordal or papillary muscle,^4^ which is how in this special case two opposing mechanisms are acting simultaneously in the rupturing of the two valves. The present case report highlights several points about these interesting cases. First, it emphasizes that significant cardiac injury can occur even with what appears to be non-penetrating blunt thoracic trauma without pericardial rupture or contusion. Second, in the case of this patient, falling from this height (five meters) had two phases. In the first phase falling from the top of the second floor to the balcony of the first floor caused a crushing blow to the sternum from a hard surface. This occurred in the diastole phase of the cardiac cycle resulting in an adrenalin surge causing rupture of aortic cusp. But the tricuspid injury occurred during the systolic phase of the cardiac cycle during the second phase of the fall from the balcony to the ground floor by falling on the left chest wall. The impact to the left chest wall from the floor, activated the initial phase of deceleration by the initial anterior displacement of the heart, leading to blood acceleration towards the valve, and because in systole, the valve is closed, energy waves produced a significant extension-tensile tug on the chordal apparatus. Fabian explains how this is quickly followed by the posterior displacement of the heart, creating a ‘reverse hemodynamic wave’ and a sudden increase in right ventricular pressure.^12^ Some of the above theories have been proved in an experimental laboratory using mathematical modeling principles by Fabian and colleagues. The other point is that delays in the correct diagnosis of cardiac valvular injury or injuries are related to associate multiple organ trauma. In this regard, perhaps a higher index of suspicion is required when patients present with a history of any severe or moderate blunt chest trauma.^12^ Initially we missed the possibility of aortic valve rupture because tricuspid valve rupture was sufficient to explain our patient’s condition. Finally, without transesophageal echocardiography, the immediate diagnosis of severe tricuspid valve or aortic regurgitation might not have been made. Ismailov explains how the decision to operate is very important and can be decided on the basis of heart function and the systemic situation and general condition of patients.^13^ If the aortic regurgitation does not impair hemodynamic stability, or if there are other lesions that may increase operative risk, delayed instead of emergency operation is advised, as this allows the surgeon to select the best conditions for intervention. Nan et al. point out that there may be an asymptomatic period for a week or more and surgeons treating these patients should be aware of the potential for rapid decompensation and sudden death and the need for an emergency operation.^14^ In West’s study, the indications for the valve replacement or repair depend on many factors: the extent of injury to the cusp; the number of cusps or commissures involved; and the ability of the surgeon to perform aortic valve repair.^15^ Parry has shown that prosthetic valve replacement is indicated in cases with a complicated tear of cusp with aortic wall or dissection or multiple lesions of the cusp or coronary ostium.^16^ In some cases where the aortic root or valve commissures are involved, replacement with a composite mechanical graft conduit should be considered.^17^ Repair methods described by Halstead such as direct suture or fresh-pericardial patch are suitable for a simple and regular rupture in one cusp.^18^ One type of rupture of the cusp received special attention by Pretre and colleagues.^19^ Pretre showed a visible tear of the non-coronary cusp during operation without any lesions of the left and right coronary cusps. But post-operative microscopic examination of the pathological valve also revealed a tear in the left cusp, which had suffered no visible laceration.^19^ Pastershank,’s study points out that repairing only the non-coronary cusp may lead to an insufficiency of the left coronary cusp in the future. So he thinks that even in the lesion of a single valve cusp, if the rupture is severe and associated with aortic valve dissection, the potential rupture of other cusps should be considered and a prosthetic valve replacement is a good, safe choice.^20^ Perlroth reported that the other mechanism of the tricuspid valve rupture beside acceleration – deceleration theory – presumably involves a severe elevation of right ventricular intracavitary pressure caused by sudden compression of the heart.^3^ As in our case, the most frequently reported injury is chordal rupture, followed by rupture of the anterior papillary muscle and leaflet tear, primarily of the anterior leaflet. Osborn revealed that rupture of the papillary muscle and severe TR causes dyspnea that typically becomes symptomatic rapidly, whereas ruptured chordae or torn leaflets and mild or moderate TR may have a more insidious onset of symptoms even though, isolated tricuspid regurgitation may initially be clinically benign. However in combination with other conditions, valve disease causes right heart failure in most patients. Traditionally, this has been the indication for surgery, which usually consists of tricuspid valve replacement. When operative intervention is unduly delayed however, irreversible right ventricular myocardial dysfunction may develop.^21^ The only patient in the Khurana series who did not have right ventricular dysfunction before the operation had the shortest interval between trauma and operation (1 month). Long-term results will likely be better if operation is performed before right ventricular function deteriorates, rather than after the onset of progressive right heart failure.^22^ Yasuura believes that this improved outcome may be particularly true if the ruptured tricuspid valve can be successfully repaired rather than replaced that relate to conservative surgery by preservation of tricuspid papillary muscle.^4^ Although the literature contains little information concerning the latest results of tricuspid valve repair, we believe results would be better with repair than with valve replacement because the geometry and function of the right ventricle are better preserved and complications inherent in prosthetic heart valves are avoided. This outcome would be analogous to the improved late results of mitral valve repair compared with valve replacement as in Galloway.^23^ However, the small number of patients in this series does not allow statistical support of this contention.

In conclusion this case represent an exceedingly rare case that simultaneously includes two valve injuries caused by the same mechanism acting separately in two different cycle of the heart i.e. systole and diastole, during falling from height.
